# Depression as a Mediator and Social Participation as a Moderator in the Bidirectional Relationship Between Sleep Disorders and Pain: Dynamic Cohort Study

**DOI:** 10.2196/48032

**Published:** 2023-07-26

**Authors:** Si Fan, Qianning Wang, Feiyang Zheng, Yuanyang Wu, Tiantian Yu, Yanting Wang, Xinping Zhang, Dexing Zhang

**Affiliations:** 1 School of Medicine and Health Management Tongji Medical College Huazhong University of Science and Technology Wuhan, Hubei China; 2 School of Public Health and Primary Care The Chinese University of Hong Kong Hong Kong Special Administrative Region Hong Kong

**Keywords:** depression, dynamic cohort, longitudinal mediation, pain, sleep disorders, social participation

## Abstract

**Background:**

Chronic pain, sleep disorders, and depression are major global health concerns. Recent studies have revealed a strong link between sleep disorders and pain, and each of them is bidirectionally correlated with depressive symptoms, suggesting a complex relationship between these conditions. Social participation has been identified as a potential moderator in this complex relationship, with implications for treatment. However, the complex interplay among sleep disorders, pain, depressive symptoms, and social participation in middle- and old-aged Asians remains unclear.

**Objective:**

This study aimed to examine the bidirectional relationship between sleep disorders and pain in middle- and old-aged Chinese and measure the role of depression as a mediator and social participation as a moderator in this bidirectional relationship through a dynamic cohort study.

**Methods:**

We used data from the China Health and Retirement Longitudinal Study across 5 years and included a total of 7998 middle- and old-aged people (≥45 years old) with complete data in 2011 (T1), 2015 (T2), and 2018 (T3). The cross-lag model was used to assess the interplay among sleep disorders, pain, depressive symptoms, and social participation. Depressive symptoms were assessed by the 10-item Centre for Epidemiological Studies Depression scale. Sleep disorders were assessed by a single-item sleep quality scale and nighttime sleep duration. The pain score was the sum of all pain locations reported. Social participation was measured using self-reported activity.

**Results:**

Our results showed significant cross-lagged effects of previous sleep disorders on subsequent pain at T2 (β=.141; *P*<.001) and T3 (β=.117; *P*<.001) and previous pain on subsequent poor sleep at T2 (β=.080; *P*<.001) and T3 (β=.093; *P*<.001). The indirect effects of previous sleep disorders on pain through depressive symptoms (β=.020; SE 0.004; *P*<.001; effect size 21.98%), as well as previous pain on sleep disorders through depressive symptoms (β=.012; SE 0.002; *P*<.001; effect size 20.69%), were significant across the 3 time intervals. Among participants with high levels of social participation, there were no statistically significant effects of previous sleep disorders on subsequent pain at T2 (β=.048; *P*=.15) and T3 (β=.085; *P*=.02), nor were there statistically significant effects of previous pain on subsequent sleep disorders at T2 (β=.037; *P*=.15) and T3 (β=.039; *P*=.24). Additionally, the mediating effects of depressive symptoms on the sleep disorders-to-pain pathway (*P*=.14) and the pain-to-sleep disorders pathway (*P*=.02) were no longer statistically significant.

**Conclusions:**

There is a bidirectional relationship between sleep disorders and pain in middle- and old-aged Asians; depression plays a longitudinal mediating role in the bidirectional relationship between them; and social participation moderates the bidirectional relationship between them directly and indirectly by affecting depression. Future interventions may consider the complex relationship between these conditions and adopt a comprehensive treatment regime.

## Introduction

Chronic pain, sleep disorders, and depression are prevalent worldwide and lead to a serious disease burden. For instance, chronic pain affects hundreds of millions of people globally, with estimates of its prevalence ranging from 10% to 40% in different countries [1-4]. It also attracted widespread attention as a global health issue. For example, Healthy People 2030 has put reducing the proportion of adults with high-impact chronic pain as one of its main goals in the United States [5]. Additionally, sleep disorders, particularly insomnia, affect over 300 million people just in China, and of those, about 30% experience one or more symptoms of insomnia, seriously affecting individuals’ physical, emotional, social, and occupational functions, and those health risks are on the rise [6]. Especially during the COVID-19 outbreak, the prevalence of insomnia increased significantly [7]. Third, depression ranks fourth in disease burden worldwide, with nearly 350 million people affected by it, which can lead to a 12.7% mortality rate [8], and other health problems often caused by depression, such as type 2 diabetes [9], cardiovascular disease [10], and suicide [11], with great burdens on families and communities [8]. Overall, chronic pain, sleep disorders, and depression often coexist and are prevalent in the general population, posing more serious health risks [12,13].

A strong link between sleep disorders and pain has been examined first in the past 2 decades. For instance, research indicates that at least 50% of insomniacs also have chronic pain [14]. Additionally, experimental studies on healthy participants have also suggested that pain levels increase after sleep restriction or interruption [15,16]. Conversely, pain has also been found to be a strong predictor of sleep disorders. For example, individuals with chronic pain often experience poor sleep quality [17,18], and it has been estimated that chronic pain patients are 18 times more likely to reach the clinical diagnostic criteria for insomnia than those without pain [19].

Second, studies have found that depressive symptoms might be bidirectionally correlated with sleep disorders and pain, respectively. Specifically, the link between depressive symptoms and sleep disorders might be bidirectional. Sleep disorders are related to maladaptive coping mechanisms that can lead to depression, especially in chronic pain patients [20]. Conversely, depressive symptoms may potentially induce sleep disorders, as evidenced by the fact that sleep disorders are the most common residual symptoms after the remission of a depressive episode [21,22]. Furthermore, there is a relationship between depressive symptoms and pain, which may be mutually reinforcing, leading to a vicious circle between them. Depression and pain symptoms follow the same descending pathways of the central nervous system. Depression may reduce the regulatory role of the descending pain system and amplify pain perception by depleting 5-hydroxytryptamine and norepinephrine [23]. Chronic pain can also induce depression through various molecular mechanisms, such as midbrain dopamine, inflammatory factors, and epigenetic modifications [24]. Thus, depressive symptoms might play a mediating effect in considering the relationship between sleep disorders and pain.

However, there have been limited high-quality studies using large-scale longitudinal data to explore the trilateral relationship among depression, sleep disorders, and pain because the existing research on the relationship between them has mainly been based on cross-sectional designs [25,26], especially in the Asian population [27]. Given the differences in geographical environment, lifestyle, race, and cultural affiliation, the prevalence of sleep deficiency [28,29] and the perception of pain [30,31] as well as the willingness to seek support for depression [32,33] may differ between older people in Asia and Western countries. Therefore, the results of Western studies may not be generalizable to Asian populations, and longitudinal studies in Asia are desperately needed to answer the question of whether sleep disorders are causally related to pain (or vice versa) and to explore the potential mediating effect of depressive symptoms on this relationship.

In addition, the primary therapeutic regimes on pain are meeting a huge challenge, and society around the globe is seeking aggressive complementary treatments. First, chronic pain might be best considered a disease. Despite clinical trials and guidelines recommending personalized, multimodal, interdisciplinary treatment approaches, chronic pain remains active [34]. Second, the primary therapeutic guidelines for sleep disorders are cognitive behavioral therapy and pharmacological treatment [35]. However, meta-analyses have shown that although cognitive behavioral therapy can improve subjective sleep quality [36], it does not improve sleep parameters defined by polysomnography [37]. Furthermore, cognitive behavioral therapy is often not available due to the limited number of trained professionals. And patients often prefer nonpharmacological treatments due to the unsatisfactory benefit-risk ratio of hypnotic drugs [38-40]. Third, it is difficult to unravel unique causal pathways due to the potential bidirectional effects between pain and sleep disorders. Measures to improve sleep may be a useful addition to pain management plans [41], which means that combined treatment of sleep disorders and chronic pain may be better than single treatment. Given that, multidisciplinary interventions that attempt to interrupt the possible vicious cycle between sleep disorders and pain may be helpful. Therefore, new explorations are needed to delve into novel approaches with better accessibility and economic effectiveness to moderate the bidirectional pathways of sleep disorders and pain.

Social participation may be a potential moderator. Previous literature suggested the potential benefits of social participation in alleviating depression, pain, and sleep disorders. Social interaction and communication during social participation may motivate mutual support, provide a sense of belonging to an individual, and to a large extent reduce social isolation [42,43], which therefore may prevent depression [44]. The biopsychosocial model of pain hypothesizes that pain is influenced by biological, psychological, and social factors. Satisfaction with one’s social role and self-perceived ability to engage in social roles during social participation are related to pain [45]. Berkman’s social relations model [46] suggests that social participation, as a downstream factor of social relations, can have a positive effect on mental health and health behaviors to promote sleep [47]. Finally, this study hypothesizes that social participation, as an accessible and cost-effective method, can moderate the bidirectional pathways of sleep disorders and pain through direct and indirect pathways.

Therefore, three main objectives of this dynamic cohort study were proposed: (1) to explore the bidirectional relationship between sleep disorders and pain; (2) to explore the longitudinal mediating role of depressive symptoms on the bidirectional relationship between sleep disorders and pain given the potentially important role of depressive symptoms; and (3) to explore the moderating effect of social participation on the bidirectional relationship between sleep disorders and pain. This study aims to provide higher levels of evidence regarding the above objectives based on a national prospective cohort study, contributing to a better understanding of the complex interplay between sleep disorders, pain, depressive symptoms, and social participation.

## Methods

### Sample and Data Collection

The data used in this study were obtained from the follow-up survey of the China Health and Retirement Longitudinal Study (CHARLS). The participants are middle- and old-aged residents. The baseline data collection for CHARLS was conducted in 2011 and included approximately 10,000 households and 17,500 individuals from 150 counties and districts and 450 villages and resident committees in 28 provinces. The follow-up surveys were conducted every 2 to 3 years, and all survey data were made public 1 year after the end of data collection.

CHARLS adopted a multistage stratified probability proportional to size sampling technique, and the detailed sampling technique can be found elsewhere [48]. All data were collected using face-to-face interviews at 3 time points, covering participants’ demographic background, family characteristics, health behavior and status, and retirement information. More detailed information about CHARLS can be found on its official website [49].

This study used the follow-up data from 2013 (T1), 2015 (T2), and 2018 (T3). The sample size is 7998 participants who are selected with a criterion of providing complete information on sleep disorders, pain, depression, and social participation in all 3 waves.

### Ethics Approval

The studies involving human participants were reviewed and approved by the Research Ethics Committees of Peking University (IRB00001052-11015). The patients or participants provided their written informed consent to participate in this study. The data for analysis were deidentified without revealing any personal identity.

### Depression

The 10-item Center for Epidemiologic Studies Depression Scale (CES-D 10) was used to measure depressive symptoms. Covering the respondent’s positive feelings, negative emotions, and physical symptoms in the past week. The item of CES-D 10 was scaled from 0 to 3, with the total score ranging from 0 to 30. Higher scores indicate more depressive symptoms. CES-D 10 has shown good internal reliability, with a Cronbach α of 0.815 [50].

### Sleep Disorders

Sleep disorders were assessed by a single-item sleep quality scale and nighttime sleep duration, which were both commonly used in previous studies [51-53]. The total score for sleep disorders was calculated as the sum of the scores for the single-item sleep quality scale and nighttime sleep duration.

Participants were asked to rate their sleep quality using the single-item sleep quality scale. The single item in the scale was “My sleep was restless,” which had a 4-point response scale ranging from “rarely or none of the time” to “most or all of the time.” The self-reported measure of sleep quality as a single item has been widely used in previous studies and has been shown to be highly correlated with the multi-item versions of the sleep quality scale [51]. Furthermore, it has been reported that the test-retest reliability coefficients of the single-item sleep quality scale reached 0.90 [54].

Nighttime sleep duration was assessed based on self-reported data in response to the question, “How many hours did you usually sleep per day on average in the past month?” According to the classification on sleep duration from the National Sleep Foundation [55] and the research from Gu et al [56], the responses were divided into 4 groups based on the number of hours slept per night and scored from 0 to 3. A score of 3 was assigned to participants who reported sleeping less than 5 hours or more than 10 hours per night; a score of 2 was assigned to those who reported sleeping between 5 and 6 hours or between 9 and 10 hours per night; a score of 1 was assigned to those who reported sleeping between 6 and 7 hours or between 8 and 9 hours per night; and a score of 0 was assigned to those who reported sleeping between 7 and 8 hours per night.

### Pain

In this study, participants were asked to report whether they experienced pain. Those who did not report experiencing pain were assigned a value of 0, while those who reported experiencing pain were asked to report their pain location using a validated body map [57], including the head, shoulders, arms, wrists, fingers, chest, stomach, back, waist, buttocks, legs, knees, ankles, toes, neck, and other parts. Each pain location was counted as 1 point, and the total pain score was the sum of all pain locations reported. These methods to determine the extent and distribution of pain are standard in population-based studies of pain and have been shown to be valid and reliable [58].

### Social Participation

Combining the definitions of social participation by previous scholars [59] and the characteristics of activity participation of Chinese seniors [60], this study adapted the definition of social participation from previous literature that emphasizes participation in activities that provide interactions with others in society or the community. Based on this adapted definition, the following types of activities were included: (1) interacting with friends; (2) playing mahjong, chess, cards, or going to the community club; (3) providing help to relatives, friends, or neighbors who do not live with you; (4) dancing, fitness, qigong, etc; (5) participating in club activities; (6) participating in volunteer activities or charity activities; (7) taking care of patients or disabled people who do not live with you; (8) going to school or attending training courses; (9) stock trading; (10) surfing the internet; and (11) other social participation that meets our definition. One social participation counted as 1 point. If the respondent answered “yes” to any of the above social participation, they were asked about the corresponding frequency (3=almost every day, 2=almost every week, and 1=not often). The total score of social participation was the cumulative score of the corresponding frequency of each individual social participation.

### Control Variable

To minimize the possibility of a third variable influence on the sleep disorders-pain relationship and to keep the parsimony of our model, a limited number of covariates related to sleep and pain were controlled. According to previous studies, all covariates are based on baseline data [61]. First, demographic control variables included age, gender (coded as 0=male and 1=female), and educational level (coded as 0=illiterate, 1=junior high school or below, and 2=high school or above). Then, lifestyle covariates included current smoking and current drinking status. The respondents were asked if they currently smoke (coded as 1=yes and 0=no) and drink (coded as 1=yes and 0=no). Interpersonal relationship covariates included marital status (coded as 1=married and 0=never married, separated, divorced, or widowed) and residence (coded as 1=rural and 0=urban). In addition, the covariates closely related to pain included “have measures been taken to relieve pain?” (coded as 1=yes and 0=no).

### Statistical Analysis

In order to maximize statistical power and minimize bias, full-information maximum likelihood estimation was conducted in Mplus (version 8.3; Muthén & Muthén). This estimation method is referred to as principled missing data handling approach, as it does not directly replace missing values but uses existing information to estimate population parameters without bias [62], and it is considered suitable for attributing missing longitudinal data, such as the data analyzed here.

The following variables were inputted into the model as continuous variables: sleep disorders, pain, depressive symptoms, social participation, and age, while the remaining variables were inputted as categorical variables. Structural equation modeling with observed variables was done to evaluate the association between sleep disorders and pain at all time points (model 1), the mediating role of depressive symptoms (model 2), and the moderating role of social participation (model 3). Thus, a full mediation structural equation model was constructed [63]. Following the suggestion of Hoyle [64], 2 fit indices were used to evaluate the model fit, including the standardized root mean square residual (SRMR) and the comparative fit index (CFI). A CFI value greater than 0.95 was considered to indicate a good model fit, while an SRMR value below 0.06 was considered to indicate a good fit, although values below 0.08 were considered to be acceptable. All mediations were tested with 5000 bootstrap iterations.

## Results

### Descriptive Statistics

The sample characteristics based on baseline data and the distribution of key variables by period are presented in Table 1. The average scores of key variables from T1 to T3 were as follows: sleep disorders (2.18, 2.20, and 2.45); depressive symptoms (7.89, 8.07, and 8.87); pain (0.94, 1.73, and 3.10); and social participation (2.01, 1.89, and 1.83), respectively.

**Table 1:** The characteristics of covariates at baseline and depressive symptoms, sleep disorders, pain, and social participation from T1 to T3 (N=7998).

Variable		2013 (T1)	2015 (T2)	2018 (T3)
Age (years), mean (SD)		58.47 (8.54)	—^a^	—
**Sex, n (%)**				
	Female	3865 (48.32)	—	—
	Male	4133 (51.68)	—	—
**Education, n (%)**				
	Illiteracy	1738 (21.73)	—	—
	Junior high school or less	5172 (64.67)	—	—
	High school or more	1088 (13.6)	—	—
**Marital status, n (%)**				
	Married	7258 (90.75)	—	—
	Not in marriage	740 (9.25)	—	—
**Residence, n (%)**				
	Urban	2991 (37.4)	—	—
	Rural	5007 (62.6)	—	—
**Smoke, n (%)**				
	Yes	2176 (27.21)	—	—
	No	5822 (72.79)	—	—
**Drink, n (%)**				
	Yes	2706 (33.83)	—	—
	No	5292 (66.17)	—	—
**Taken measures to relieve pain, n (%)**				
	Yes	1508 (18.85)	—	—
	No	6490 (81.15)	—	—
Pain, mean (SD)		0.94 (2.05）	1.73 (3.41）	3.10 (3.79）
Depressive symptoms, mean (SD)		7.89 (5.75）	8.07 (6.38）	8.87 (6.52）
Sleep disorders, mean (SD)		2.18 (1.93）	2.20 (1.95）	2.45 (1.98）
Social participation, mean (SD)		2.01 (2.37)	1.89 (2.41)	1.83 (2.38)

^a^Not available.

### The Relationship Between Sleep Disorders and Pain

**Figure 1:**
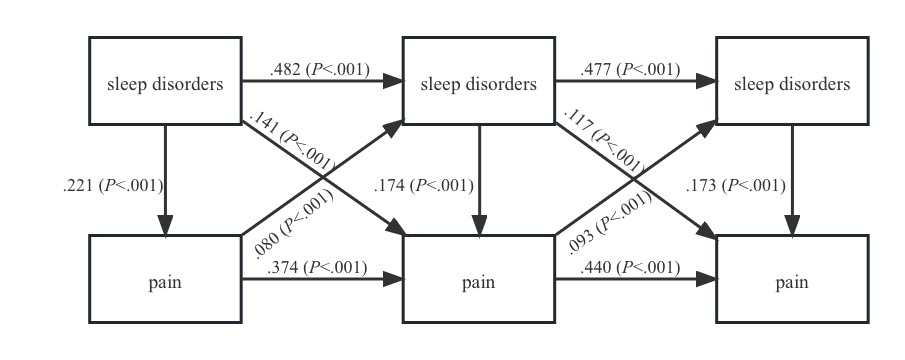
Cross-lagged panel model of the bidirectional relationship between sleep disorders and pain.

Figure 1 depicts the cross-lagged model of the bidirectional relationship between sleep disorders and pain (model 1). After controlling for covariates, model 1 fitted the data adequately (CFI=0.919; SRMR=0.045). Sleep disorders at each time point were positively related to themselves over time, as was pain, as would be expected. The cross-lagged effects of previous sleep disorders on pain at T2 (β=.141; *P*<.001) and T3 (β=.117; *P*<.001) as well as pain on subsequent sleep disorders at T2 (β=.080; *P*<.001) and T3 (β=.093; *P*<.001) were significant. These results support our hypothesis that sleep disorders and pain are bidirectionally related.

### The Longitudinal Mediating Role of Depressive Symptoms

**Figure 2:**
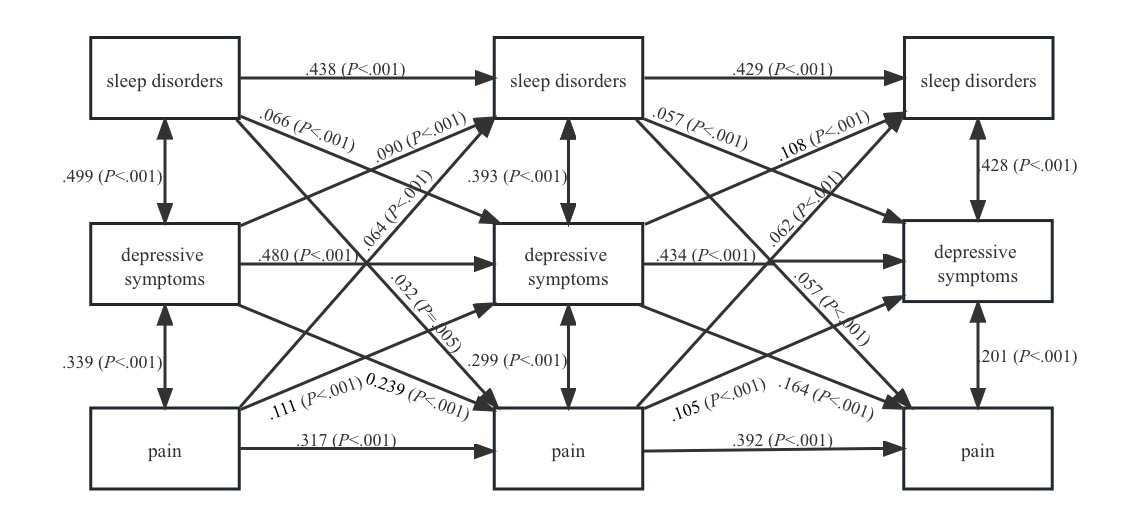
Longitudinal mediating effect of depressive symptoms in the bidirectional relationship between sleep disorders and pain.

As Figure 2 presents, after adding 2 indirect paths that shared depressive symptoms as a potential mediator, model 2 still had a good fit to the data (CFI=0.983; SRMR=0.045). At a significance level of 0.01, we found that the cross-lagged effects of sleep disorders on subsequent pain at T2 (β=.032; *P*=.005) and T3 (β=.057; *P*<.001), as well as previous pain on subsequent sleep disorders at T2 (β=.064; *P*<.001) and T3 (β=.062; *P*<.001), were significant but slightly reduced in size compared to those in model 1.

We found 2 specific indirect paths from the depressive symptoms. First, higher previous sleep disorders predicted worse subsequent depressive symptoms (β=.066; *P*<.001), and worse previous depressive symptoms predicted higher amounts of subsequent pain (β=.164; *P*<.001). Second, higher previous pain predicted worse subsequent depressive symptoms (β=.111; *P*<.001), and worse previous depressive symptoms predicted higher amounts of subsequent sleep disorders (β=.108; *P*<.001). To further test the longitudinal mediation, we used a 95% CI in Mplus (Table 2). Effect sizes of the longitudinal mediations were computed using MacKinnon’s formula for calculating the mediated percentage, which is the indirect effect divided by the total effect (Table 2). The indirect effects of previous sleep disorders on pain through depressive symptoms (β=.020; SE 0.004; *P*<.001; effect size 21.98%), as well as previous pain on sleep disorder through depressive symptoms (β=.012; SE 0.002; *P*<.001; effect size 20.69%), were significant across the 3-time intervals. Both effect sizes fell in the medium range on the basis of Cohen’s guidelines, suggesting that these mediations captured significant covariation over time.

**Table 2:** Statistical results of longitudinal mediating effect of depressive symptoms in the bidirectional relationship between sleep disorders and pain.

Model 2	β	SE	*P* value	Effect size (%)^a^
T1 sleep disorders to T2 depressive symptoms to T3 pain	.020	0.004	<.001	21.98
T1 pain to T2 depressive symptoms to T3 sleep disorders	.012	0.002	<.001	20.69

^a^Effect size is the proportion mediated, which is calculated by dividing the indirect effect by the total effect.

### The Moderating Effect of Social Participation

**Figure 3:**
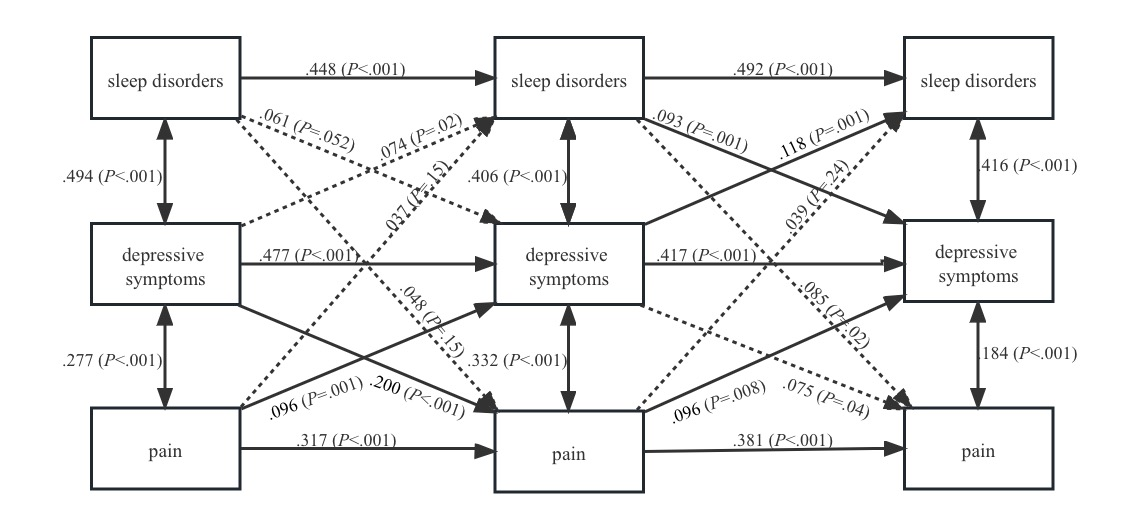
Moderating effect of social participation in the relationship among sleep disorders, pain, and depressive symptoms.

To test our third hypothesis, we examined whether social participation could moderate the bidirectional relationship between sleep disorders and pain in model 3. The results of model 3 are shown in Figure 3 and Table 3 and fit the data well (CFI=0.983; SRMR=0.045). We found support for this prediction in participants with high levels of social participation (greater than the mean). As shown in Figure 3, social participation directly moderates the bidirectional relationship between sleep disorders and pain. This was demonstrated by the lack of significant cross-lagged effects between sleep disorders and pain in participants with high levels of social participation, at a significance level of .01. Specifically, we did not observe statistically significant effects of sleep disorders on subsequent pain at T2 (β=.048; *P*=.15) and T3 (β=.085; *P*=.02), nor did we observe statistically significant effects of previous pain on subsequent sleep disorders at T2 (β=.037; *P*=.15) and T3 (β=.039; *P*=.24).

**Table 3:** Statistical results of longitudinal mediating effect of depressive symptoms in the bidirectional relationship between sleep disorders and pain in participants with high levels of social participation.

Model 3	β	SE	*P* value	Effect size (%)^a^
T1 sleep disorders to T2 depressive symptoms to T3 pain	.010	0.007	.14	4.95
T1 pain to T2 depressive symptoms to T3 sleep disorders	.016	0.007	.02	28.57

^a^Effect size is the proportion mediated, which is calculated by dividing the indirect effect by the total effect.

Using a 95% CI in Mplus (Table 3), we found that depressive symptoms no longer mediated the sleep disorders-to-pain pathway (*P*=.14) and the pain-to-sleep disorders pathway (*P*=.02) in participants with high levels of social participation. This indicated that social participation indirectly moderated the bidirectional relationship between sleep disorders and pain by affecting depression.

## Discussion

### Principal Findings

In summary, the dynamic cohort study based on the longitudinal data in middle- and old-aged Asians demonstrates a reciprocal relationship between sleep disorders and pain, and the prospective reciprocal relationship is partially mediated by depressive symptoms. The reciprocal relationship between sleep disorders and pain is also moderated by social participation directly and indirectly by affecting depression.

Previous research already showed that sleep disorders, pain, and depressive symptoms are cross-sectionally related. Although there are longitudinal studies and even a randomized controlled study [65] in Western countries, they only suggest that sleep disorders and pain interact in a bidirectional manner. Despite some cross-sectional studies demonstrating the indirect path from pain to sleep disorders through depression symptoms [26] and the reverse influence [25], the data are cross-sectional and correlational, meaning that causality cannot be inferred and firm conclusions about mediation effects cannot be drawn. To our knowledge, there has been 1 cohort study [66], but depressive symptoms were assessed during the same wave as sleep disorders, making it uncertain that they were indeed a consequence of sleep disorders. Our study explores the basic mechanism of the mutual relationship between depression, sleep disorders, and pain and provides higher-level evidence by large-scale longitudinal data. Our findings not only strengthen the bidirectional relationship between sleep disorders and pain and the longitudinal mediating effect of depression symptoms in this bidirectional relationship, but also explore the social participation as an accessible and feasible moderation measure to interrupt the vicious cycle of sleep disorders and pain directly and indirectly by affecting depression, which will provide inspiration for the treatment of sleep disorders and pain. Our study contributes to the literature by examining the prospective relationship between sleep disorders, pain, depression, and social participation in a large-scale cohort study. The advantages of our study include large sample size, prospective design, and nationally representative data [67].

Collectively, our findings from a large cohort of middle- and old-aged Chinese population support the bidirectional relationship between sleep disorders and pain, with a stronger effect of sleep disorders on pain at T2 (β=.141; *P*<.001) and T3 (β=.117; *P*<.001) compared with the effect of pain on sleep disorders at T2 (β=.080; *P*<.001) and T3 (β=.093; *P*<.001). These findings are in accordance with previous research conducted in Western countries. For instance, a study found that poor baseline sleep quality predicted higher pain intensity at 6 months (β=.18; 95% CI 0.07-0.30), and vice versa (β=.14; 95% CI 0.01-0.26) [68]. Additionally, a randomized controlled trial revealed that changes in sleep complaints at 3 months significantly predicted changes in pain at 12 months (β=.29; *P*<.001), and to a lesser extent, changes in pain predicted changes in sleep (β=.15; *P*<.05) [65], which is consistent with our study in terms of the direction and strength of the bidirectional relationship between sleep disorders and pain. However, a study of Western emerging adults (18-25 years old) partly argued against the long-term bidirectional relationship, suggesting that sleep disorders significantly increased chronic pain (β=.15; *P*<.05) [66]. In comparison, we further found a slightly weaker but significant longitudinal effect of pain on sleep disorders, which may be due to the different age stages and races of the population.

There is evidence that emotional disturbances and negative emotions such as depression can largely explain the relationship between pain and sleep disorders. For instance, a study found that depression partially mediated the effects of pain, substantiating an indirect path from pain to sleep disturbance through depressive symptoms, and this indirect effect (β=.20; SE 0.07) accounted for 37.46% of the total effects in the model tested [26]. Another study showed that sleep disorders have a strong positive association with depressive symptoms (β=.67; 95% CI 0.57-0.75; *P*<.001), which in turn has a moderate positive association with pain severity (β=.36; 95% CI 0.19-0.51; *P*<.001), and the mediating effect of depressive symptoms was significant (β=.24; SE 0.052) [25]. In addition, a cohort study found that sleep disorders indirectly affect the severity of abdominal pain through depressive symptoms (β=.05; 95% CI 0.01-0.09; k^2^=0.06) [66]. In agreement with these studies, our results support the mediating role for depression symptoms in the bidirectional relationship between sleep disorders and pain, confirming an indirect pathway from pain to sleep disorders through depressive symptoms and the reverse influence. Therefore, previous findings suggesting a causal link between sleep disorders and pain may be, in part, due to the effects of depressive symptoms. Our study contributes to the current understanding of the relationship between sleep disorders, pain, and depression in the Asian population, providing valuable insights where there was previously a gap in knowledge.

In the middle- and old-aged population with high levels of social participation, we identified that social participation moderated the bidirectional relationship between sleep disorders and pain directly and indirectly by affecting depression. This suggests that for individuals with pain, higher levels of social participation are associated with a reduced likelihood of experiencing sleep disorders. Similarly, for individuals with sleep disorders, higher levels of social participation are associated with a reduced likelihood of experiencing pain. This is a new finding, as previous related studies only suggested that social participation has potential benefits in alleviating depression, pain, and sleep disorders. For instance, social participation has a significant negative effect on the depressive symptoms of the older adults, whether they are empty-nest older adults (β=–2.404; *P*<.001) or non–empty-nest older adults (β=–1.957; *P*<.001) [44]. Additionally, high-frequency social participation, such as participating in interest groups (β=–.59; *P*<.01), community-related organizations (β=–.41; *P*<.05), sports (β=–.39; *P*<.05) can have a positive effect on mental health and health behaviors to promote sleep [47]. Moreover, satisfaction with their social roles during social participation (β=–.16; *P*<.01) and self-perceived ability to participate in social roles are related to pain (β=–.19; *P*<.001) [45]. However, there is little systematic study on the role of social participation in the cyclical relationship between sleep disorders, depression, and pain. This study reveals that social participation can serve as a practical and cost-effective strategy to moderate the bidirectional relationship between sleep disorders and pain directly and indirectly by affecting depression, which has potential implications for their treatment. Additionally, social participation may also moderate the relationship between sleep disorders and pain by affecting circadian rhythms, as suggested by the literature. For example, social participation in activities such as dancing and fitness can modulate the molecular clock in skeletal muscle, affecting both the amplitude and phase of circadian rhythms [69]. Disrupted circadian rhythms have direct effects on both sleep disorders and pain, as multiple clinical and foundational science studies have reported that circadian rhythm disruption can directly alter pain thresholds [70]. Moreover, disrupted circadian rhythms can lead to undesirable or irregular timing of sleep, exacerbating sleep disorders [71].

Future research could investigate the effects of various types of social participation with different durations, frequencies, and timings on circadian rhythms. Future research could compare the impacts of overnight activities such as playing mahjong or caring for disabled family members with daytime activities like helping neighbors or engaging in volunteer work. Future research could also investigate the effects of various types of social participation with different durations, frequencies, and timings on the relationship. Such research could shed light on the intricate relationship between social participation, circadian rhythms, sleep disorders, and pain.

### Limitations

Although this study indicates some interesting relationship, there are limitations to the interpretation of the results. First, the self-reported nature of the data means that results must be interpreted with caution. There may be a tendency to over- or underestimate symptoms. Second, data were not collected on specific pain diagnoses. Different potential diseases and causes may play a role in the relationship between pain and sleep, so it would be useful in the future to collect such data for subgroup analysis. Third, medical information such as the disease itself and other comorbidities, as well as the severity of symptoms and medication records, was not collected and therefore could not be controlled for in our analysis. For example, some analgesic compounds can lead to sleep fragmentation and even insomnia [72]. Future research should consider the effects of medications on sleep structure as well as emotional and physical states. Fourth, due to our inability to incorporate potential confounders into the analysis or adjust for them, such as stressful life events, family history of mental disorders or genetic characteristics, as well as family characteristics, health behaviors and status, and retirement information, residual confounding may exist. Future studies should consider these limitations and attempt to address them to provide a more comprehensive understanding of this complex relationship. In addition, categorizing risk factors for sleep, such as susceptibility, precipitating, and persistent factors, to better identify associated factors will provide more targeted recommendations for the management of pain, sleep disorders, and other symptoms.

### Conclusions

In conclusion, our longitudinal cohort found a bidirectional relationship between sleep disorders and pain in middle- and old-aged Asians; depression plays a longitudinal mediating role in the bidirectional relationship between them; and social participation moderated the bidirectional relationship between them directly and indirectly by affecting depression. In future research, the moderation mechanism of social participation will be a focus with high interest, which will provide further insights into the nonpharmacological treatment and care of sleep disorders and pain. Overall, future interventions may consider the complex relationship of these conditions and adopt a comprehensive treatment regime.

## Abbreviations

CES-D 10: 10-item Center for Epidemiologic Studies Depression Scale
CFI: comparative fit index
CHARLS: China Health and Retirement Longitudinal Study
SRMR: standardized root mean square residual

## Data Availability

The CHARLS data set is publicly available on its official website [49]. The data analyzed in our study can be obtained from the corresponding author upon request.
